# The impact of intravenous thrombolysis on outcome of patients with acute ischemic stroke after 90 years old

**DOI:** 10.1186/s12877-016-0331-1

**Published:** 2016-08-25

**Authors:** S. Sagnier, P. Galli, M. Poli, S. Debruxelles, P. Renou, S. Olindo, F. Rouanet, I. Sibon

**Affiliations:** 1 Unité Neuro-vasculaire, Pôle de Neurosciences Cliniques, Hôpital Pellegrin, CHU Bordeaux, UnitéBordeaux Segalen, 33076, Bordeaux, France; 2 Université Bordeaux Segalen, Bordeaux, France

**Keywords:** Intravenous thrombolysis, Very old patients, Mismatch, Functional prognosis, Hemorrhagic transformation, Post-stroke complications

## Abstract

**Background:**

Age increases the risk of mortality and poor prognosis following stroke. The benefit of intravenous thrombolysis in very old patients remains uncertain. The purpose of the study was to evaluate the efficacy and safety of thrombolysis in very old patients considering their perfusion-imaging profile.

**Methods:**

We conducted a retrospective study including patients older than 90 y.o. admitted for an acute ischemic stroke. A computed tomography perfusion-imaging (CTP) was performed in patients who received thrombolysis. Primary outcome was the functional status at 3 months, assessed by the modified Rankin scale (mRS). Secondary outcomes were the rate of hemorrhagic transformations, duration of hospitalization and the rate of death in the first 7 days. Patients receiving thrombolysis were compared with an age-matched group of non-thrombolysed patients.

**Results:**

78 patients were included (31 % male, aged 92 ± 1.7 y.o). 37 patients received thrombolysis and among them, 30 had CTP with a mismatch. The three months mRS was not significantly different in the two groups (mRS 0–2: 5 % and 7 % in the thrombolysed and non-thrombolysed group, respectively). Hemorrhagic transformations were more frequent in the thrombolysed group (54 % versus 12 %, *p* = 0.002) and symptomatic intracranial hemorrhage tended to be associated with mRS at three months and death in the first 7 days. Duration of hospitalization was longer in the thrombolysed group (10 days ± 12 versus 7 days ± 9, *p* = 0.046).

**Conclusions:**

Patients who received thrombolysis did not have a better functional prognosis than non-thrombolysed patients.

**Electronic supplementary material:**

The online version of this article (doi:10.1186/s12877-016-0331-1) contains supplementary material, which is available to authorized users.

## Background

Stroke care in older people is becoming a public health problem given the increased ageing of the population [1]. Indeed, the incidence of stroke increases with age and about 30 % of stroke patients are older than 80 years old [2]. It is widely admitted that oldest patients have a worse post-stroke functional outcome and a higher rate of death [3, 4]. Mortality at three months increases by 72 % whereas probability of good outcome decreases by 25 % every ten years [5].

The benefit of intravenous thrombolysis (IV-tPA) in ischemic stroke (IS) patients has been widely demonstrated [6, 7] but most of the studies excluded patients older than 80, therefore potentially limiting thrombolysis in this population. Several observational studies found that, compared to younger patients, tPA infusion to patients older than 80 y.o. was associated with higher functional dependency [3, 4, 8, 9]. While no significant increase of hemorrhagic transformation was observed in these older patients, this worse functional outcome seemed to be related to a worse pre-stroke functional status, a higher clinical severity at baseline, and more frequent post-stroke complications during the acute (pneumonia, heart failure). Despite the less favorable outcome of older patients, recent studies have suggested that IV-tPA could improve the functional prognosis of patients aged between 80 and 90 y.o. [10, 11]. However, old age remains a limitation for using IV-tPA for many physicians [12, 13]. That is even more true in the subgroup of very old patients, those aged over 90 y.o., for whom available data are scarce. In this population, few studies [4, 10] failed to identify a benefit of IV-tPA on functional outcome or mortality at three months. None of these studies have evaluated the potential benefit of IV-tPA depending on the neuroradiological imaging pattern at baseline. While still a matter of debate, some studies have suggested that a perfusion mismatch was associated with a better post-stroke functional prognosis among patients receiving IV-tPA [14]. The aim of our study was to evaluate the influence of IV-tPA on the three months functional outcome in a population of patients aged over 90 y.o., considering their perfusion-imaging profile.

## Methods

### Study design and patients

Patients older than 90 y.o. admitted in the emergency department of the Bordeaux University hospital for an acute IS between October 2012 and June 2015 were retrospectively included. IS was diagnosed by a stroke neurologist based on clinical and imaging data. Inclusion criteria were an admission in the first twelve hours following symptoms onset, a pre-stroke modified Rankin scale (mRS) < 4, an absence of pre-stroke severe cognitive impairment, an available computerized neurovascular medical record and a set of brain images including a computed tomography (CT) and angio-CT of the cervical and intracranial arteries at baseline and a follow-up brain imaging (brain magnetic resonance imaging [MRI] or CT-scan in case of MRI contraindication) within 72 h, confirming the IS. Moreover, for patients receiving IV-tPA, a CT-perfusion (CTP) imaging at baseline had to be available. IV-tPA was administered according to the ESO guidelines [15] at the dose of 0.9 mg/kg until 4.5 h after symptoms onset in absence of usual contraindications. Patients admitted after the first 4.5 h were not thrombolysed and CTP were not performed. They formed a non-thrombolysed age-matched group for comparisons with the thrombolysed group. Patients with transient ischemic attack were excluded from the study.

### Demographic and clinical data

Demographic and clinical data were recorded for each patient from their computerized medical record. Pre-stroke functional and cognitive status were evaluated using the pre-stroke mRS [16] and the Informant Questionnaire on Cognitive Decline in the Elderly (IQCODE) [17]. Stroke severity at baseline and at 24 h was evaluated by a stroke neurologist using the NIHSS [18]. The delay between symptom onset and IV-tPA administration, the duration of hospitalization and post-stroke in-hospital complications (delirium, mood disorders, swallowing disorders, seizure, acute coronary syndrome, pulmonary and urinary tract infection) were recorded. Stroke subtypes were defined according to the TOAST classification (Trial of Org 10172 in Acute Stroke Treatment) [19].

A clinical assessment was realized at three months post-stroke by a neurologist or a geriatrist and functional outcome was evaluated using the mRS. A three months mRS ranged 0 to 2 (i.e. alive and independent) indicated favorable outcome. The destination at the end of hospitalization (home, rehabilitation center, other medical department or nursing home) was also recorded.

### Imaging acquisition and analysis

Images were acquired at the Bordeaux University hospital on a 64-slice CT (General Electric Optima 660). Analysis was performed by two neurologists who had experience in CT and MRI examination (GP and SS). CTP source images were loaded on an Advantage workstation Volume Share 5 (General Electric Healthcare). Perfusion maps were automatically generated including mean transit time (MTT), cerebral blood volume (CBV) and cerebral blood flow (CBF) maps, using CT Perfusion 4 (General Electric Healthcare), a commercially available software based on a delay-insensitive algorithm that is unaffected by delay between arterial input and tissue curves. A mismatch was visually defined by a MTT – CBV difference greater than 20 % [20]. The Alberta Stroke Program Early CT score (ASPECTS) [21] was calculated on the non-contrast CT. ASPECTS is a score ranged from 0 to 10 and reflects the infarct burden (a low score meaning a more severe infarct). The presence and location of intracranial arterial occlusion were identified on CT-angiography of cervical and intracranial arteries. Hemorrhagic transformation was assessed on follow-up brain imaging according to the radiological criteria of ECASS I [7]: hemorrhagic infarction (HI 1 and 2) or parenchymal hemorrhage (PH 1 and 2). Symptomatic intracranial hemorrhage was defined by the presence of a hemorrhagic transformation accompanied by neurological deterioration reflected by a worsening of the NIHSS at 24 h of at least two points. Microbleeds were assessed on T2* MRI sequences according to the STRIVE criteria [22] and were classified depending on their number: absence, between 1 and 5, and > 5.

### Outcomes

Primary outcome was the mRS at three months. Secondary outcomes were the presence of a hemorrhagic transformation on the follow-up brain imaging, duration of hospitalization and death in the first 7 days.

### Statistical analysis

Qualitative variables were expressed as numbers and percentages, and quantitative variables as means and standard deviations (SD). Comparisons of quantitative variables were assessed using a *t*-test or a Wilcoxon test after verifying the conditions of application, and comparisons of qualitative variables were assessed using a Chi^2^ test. Variables associated with thrombolysis were included in a bivariate analysis with primary and secondary outcomes as dependent variables, using a linear regression model. A multivariate analysis was performed for each outcome using multiple linear regressions, and including all variables with *p* < 0.1 in the bivariate analysis. A *p*-value < 0.05 was considered significant. Statistical analysis were performed with R software version 3.2.2.

## Results

### Patients

**Fig. 1:**
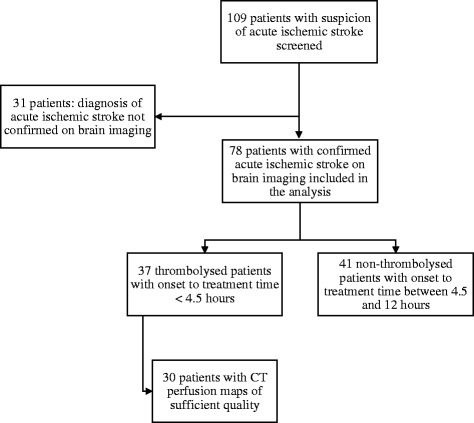
Patient’s flow chart

Seventy -eight patients were included (Fig. 1), 35 % male, aged 91.9 ± 1.7 (mean ± SD) y.o. Demographic data are described in Table 1. 37 patients received IV-tPA. Among them, 30 patients had perfusion maps of sufficient quality to be analyzed and all of them had a mismatch. 41 patients did not receive IV-tPA. All of the patients were hospitalized in an intensive care unit, and those who did not receive IV-tPA had a 250 mg bolus of Aspirin in the first twelve hours. Patients who were taking anticoagulants before stroke also received aspirin as blood biological tests showed no efficacy of their treatment. The two groups were similar in terms of age, pre-stroke functional and cognitive status. Patients who received IV-tPA had significantly more history of hypercholesterolemia (41 % versus 20 %, *p* = 0.04) and more statins as current treatment (32 % versus 10 %, *p* = 0.01). Their mean total cholesterol level at baseline did not differ (1.9 g/L ± SD 0.3 versus 1.9 g/L ± SD 0.4, *p* = 0.7, in the thrombolysed and non-thrombolysed group, respectively).

**Table 1:** Demographic data

	Thrombolysed	Non-thrombolysed	p
	*N* = 37	*N* = 41	
Male, n (%)	17 (46)	10 (24)	0.05
Age, mean (SD)	91.7 (1.5)	92 (1.8)	NS
Vascular risk factors, *n* (%)			
Hypertension	33 (89)	35 (85)	NS
Hypercholesterolemia	15 (41)	8 (20)	0.04
Diabetes mellitus	5 (14)	4 (10)	NS
Current smoking	1 (3)	0	NS
Medical history, *n* (%)			
Atrial fibrillation	12 (32)	9 (22)	NS
Ischemic heart disease	3 (8)	3 (7)	NS
Heart failure	3 (8)	5 (12)	NS
Stroke or transient ischemic attack	6 (16)	10 (24)	NS
Current treatment, *n* (%)			
Antiplatelets	19 (51)	20 (49)	NS
Oral anticoagulants	2 (5)	6 (15)	NS
Antihypertensive drugs	28 (76)	28 (68)	NS
Statins	12 (32)	4 (10)	0.01
Antidepressant drugs	6 (16)	3 (7)	NS
Pre-stroke status			
IQCODE, mean (SD)	3.2 (0.7)	3.3 (0.5)	NS
mRS > 1, *n* (%)	14 (38)	19 (46)	NS
At home, *n* (%)	32 (87)	29 (71)	NS
Nursing home, *n* (%)	5 (14)	12 (29)	NS

*SD* Standard deviation, *IQCODE* Informant Questionnaire on Cognitive Decline in the Elderly, *mRS* modified Rankin Scale, *NS* Non-significant

Clinical and radiological data at baseline are presented in Table 2. Stroke severity was not significantly different between the two groups (mean NIHSS 16 ± SD 7 in the thrombolysed group versus 13 ± 6 in the non-thrombolysed group, *p* = 0.06). The ASPECTS measured on non-contrast CT was similar in the two groups. Intracranial occlusion was significantly more frequent in the thrombolysed group (73 % versus 34 %, *p* = 0.002) with a predominance of M1 and M2 occlusion of the middle cerebral artery.

**Table 2:** Acute clinical and radiological status, post-stroke outcome and stroke mechanisms

	Thrombolysed	Non-thrombolysed	p
	*N* = 37	*N* = 41	
Acute clinical status, mean (SD)			
NIHSS at baseline	16 (7)	13 (6)	0.06
NIHSS at 24 h	15 (8)	12 (7)	NS
Systolic blood pressure (mmHg)	151 (28)	164 (25)	0.03
Diastolic blood pressure (mmHg)	79 (18)	85 (22)	NS
Temperature (Celsius degree)	36.4 (0.6)	36.6 (0.6)	NS
Capillary blood glucose level (g/L)	1.2 (0.3)	1.2 (0.4)	NS
Onset to needle time, mean (SD)	168 min (48)	-	-
Imaging parameters			
Mismatch, *n* (%)	30 (81)	-	-
Non-contrast CT ASPECTS, mean (SD)	8.3 (2.2)	8.4 (3.3)	NS
Intracranial occlusion, *n* (%)	27 (73)	14 (34)	0.002
M1	12 (45)	7 (50)	NS
M2	10 (37)	3 (22)	0.02
M3	2 (7)	1 (7)	NS
ACA	1 (4)	1 (7)	NS
PCA	2 (7)	1 (7)	NS
VB	0	1 (7)	NS
Microbleeds, *n* (%)	(*n* = 25)	(*n* = 24)	
1 – 5	6 (24)	6 (25)	NS
>5	1 (4)	0	NS
Post-stroke complications, *n* (%)			
Delirium	11 (30)	3 (7)	0.01
Mood disorder	3 (8)	2 (5)	NS
Swallowing disorder	14 (38)	4 (10)	0.003
Seizure	0	1 (2)	NS
Ischemic heart attack	1 (3)	3 (7)	NS
Pulmonary infection	12 (32)	6 (15)	NS
Urinary infection	3 (8)	1 (2)	NS
Hemorrhagic transformation, n (%)	20 (54)	5 (12)	0.002
HI 1 and 2	8 (40)	4 (80)	NS
PH 1 and 2	12 (60)	1 (20)	0.003
Symptomatic intracranial hemorrhage, n (%)	8 (22)	0	0.08
Duration of hospitalization, mean (SD)	10 days (12)	7 days (9)	0.046
mRS 0–2 at 3 months, n (%)	2 (5)	3 (7)	NS
Post-stroke outcome, n (%)			
Death ≤ 7 days	8 (22)	4 (10)	NS
Death > 7 days	6 (16)	6 (15)	NS
Return to home	5 (14)	7 (17)	NS
Rehabilitation center	10 (39)	7 (17)	NS
Other medical department	8 (22)	15 (37)	NS
Nursing home	3 (8)	10 (24)	NS
Stroke subtypes (TOAST), n (%)			
Large artery atherosclerosis	3 (8)	10 (24)	NS
Cardioembolic (AF)	27 (73)	24 (59)	NS
Small vessel disease	0	0	-
Other	0	1 (2)	NS
Undetermined	7 (19)	6 (15)	NS

*NIHSS* National Institute of Health Stroke Scale, *ASPECTS* Alberta Stroke Program Early CT score, *M1 - M2 - M3* segments of middle cerebral artery, *ACA* anterior cerebral artery, *PCA* posterior cerebral artery, *VB* vertebral-basilar arteries, *HI* hemorrhagic infarction, *PH*, parenchymal hemorrhage, *AF* atrial fibrillation, *NS* non-significant

### Post-stroke outcomes and complications

Primary and secondary outcomes are described in Table 2. The functional prognosis at three months was poor but similar in the two groups (7 % and 14 % mRS ≤ 2 at three months in the thrombolysed and non-thrombolysed group, respectively). Duration of hospitalization tended to be longer in the thrombolysed group (10 days ± 12 versus 7 days ± 9, *p* = 0.046). The mortality rate in the first seven days was higher in patients receiving IV-tPA but not significantly different from the other group (22 % versus 10 %). The most frequent post-stroke complications were swallowing disorders (38 %), pulmonary infections (32 %) and delirium (30 %). The rates of swallowing disorders and delirium were significantly higher in patients receiving IV-tPA (*p* = 0.003 and 0.01, respectively). The rate of hemorrhagic transformations was also higher in the thrombolysed group (54 % versus 17 %, *p* = 0.002), with a predominance of parenchymal hemorrhages and a trend to more symptomatic intracranial hemorrhage (40 % in the thromboysed group and no symptomatic intracranial hemorrhage in the non-thrombolysed group, *p* = 0.08).

In bivariate analyses (Table 3), hemorrhagic transformation was associated with NIHSS at baseline and at 24 h (β = 0.02, *p* = 0.01 and β = 0.03, *p* = 0.003, respectively), presence of an intracranial occlusion (β = 0.4, *p* = 0.002) and thrombolysis (β = 0.4, *p* = 0.002). mRS at three months was also associated with NIHSS at baseline (β = 0.08, *p* = 0.008) and at 24 h (β = 0.09, *p* < .001), presence of an intracranial occlusion (β = 1.04, *p* = 0.01), together with hemorrhagic transformation (β = 0.8, *p* = 0.04) and symptomatic intracranial hemorrhage (β = 1, *p* = 0.03). Death in the first seven days was also associated with symptomatic intracranial hemorrhage (β = 0.6, *p* < .001) together with NIHSS at baseline and at 24 h. There was no significant association with duration of hospitalization. Additional bivariate analyses were performed with swallowing disorders and delirium as dependent variables. Swallowing disorders were associated with NIHSS at 24 h (β = 0.02, *p* = 0.02), and thrombolysis (β = 0.3, *p* = 0.003). Delirium was also associated with thrombolysis (β = 0.2, *p* = 0.01) and the presence of an intracranial occlusion (β = 0.2, *p* = 0.047).

**Table 3:** Bivariate analyses of associations between pre-stroke and stroke characteristics and outcomes (linear regressions)

	mRS at three months		Hemorrhagic transformation		Duration of hospitalization		Death ≤ 7 days	
	Estimate β (SE)	p	Estimate β (SE)	p	Estimate β (SE)	p	Estimate β (SE)	p
Age	−0.01 (0.1)	0.9	0.02 (0.04)	0.6	0.9 (0.7)	0.2	−0.03 (0.02)	0.3
Male	−0.05 (0.05)	0.9	0.03 (0.1)	0.8	3.4 (2.5)	0.2	−0.1 (0.09)	0.1
Hypercholesterolemia	−1.1 (0.4)	0.009	0.003 (0.1)	0.9	−1.9 (2.6)	0.5	−0.2 (0.09)	0.02
Statins	−1 (0.5)	0.06	−0.06 (0.1)	0.7	−0.1 (3)	0.9	−0.1 (0.1)	0.3
Systolic blood pressure at baseline	−0.0004 (0.008)	0.9	−0.003 (0.002)	0.2	−0.0003 (0.05)	0.9	−0.003 (0.002)	0.07
NIHSS at baseline	0.08 (0.03)	0.008	0.02 (0.009)	0.01	0.2 (0.2)	0.4	0.02 (0.006)	0.002
NIHSS at 24 h	0.09 (0.02)	<.001	0.03 (0.008)	0.003	0.07 (0.2)	0.7	0.03 (0.005)	<.001
Intracranial occlusion	1.04 (0.4)	0.01	0.4 (0.1)	0.002	2.9 (2.5)	0.2	0.03 (0.08)	0.7
Thrombolysis	0.2 (0.4)	0.6	0.4 (0.1)	0.002	2.7 (2.4)	0.3	0.1 (0.08)	0.2
Hemorrhagic transformation	0.8 (0.4)	0.04	-	-	2.3 (2.8)	0.4	0.1 (0.09)	0.1
Symptomatic intracranial hemorrhage	1 (0.4)	0.03	-	-	−5.2 (6.4)	0.4	0.6 (0.2)	<.001
Cardioembolic stroke subtype (AF)	0.9 (0.4)	0.053	−0.03 (0.1)	0.8	4.6 (2.5)	0.07	0.07 (0.09)	0.4
Large artery atherosclerosis stroke subtype	−0.09 (0.6)	0.9	−0.05 (0.2)	0.8	−4.8 (3.2)	0.1	−0.09 (0.1)	0.4

*SE* standard error, *AF* atrial fibrillation

In multivariate analyses (Table 4), only the NIHSS at 24 h (β = 0.03, *p* = 0.006) and the presence of an intracranial occlusion (β = 0.3, *p* = 0.02) remained associated with hemorrhagic transformation. No significant association persisted with mRS at three month, death in the first seven days (see Additional file 1), swallowing disorders and delirium.

**Table 4:** Predictors of hemorrhagic transformation in multivariate analysis (multiple linear regressions)

	Hemorrhagic transformation	
	Estimate β (SE)	p
NIHSS at baseline	−0.002 (0.01)	0.8
NIHSS at 24 h	0.03 (0.01)	0.006
Intracranial occlusion	0.3 (0.1)	0.02
Thrombolysis	0.03 (0.1)	0.8

## Discussion

The main result of this study is the absence of significant difference on functional outcome at three months between patients older than 90 y.o. receiving and not receiving IV-tPA for an acute IS. In line with previous studies, this result suggests poor outcome in this very elderly population treated with IV-tPA for acute IS [4, 10]. However, a recent sub-group analysis of the Third International Stroke Trial (IST-3) reported a good outcome in 111 patients > 90 y.o. treated with alteplase versus 98 control patients, and this good outcome was not significantly different from the other groups [23]. Our different results might be explained by the lack of power of our study.

Interestingly, our results were observed despite a favorable neuroimaging pattern on CTP imaging with the presence of a MTT-CBV mismatch in all available perfusion maps of the patients receiving IV-tPA and presence of a high ASPECT score [24, 25]. The impact of CTP imaging profile in patients receiving IV-tPA is not clearly established in the literature. This result suggests that even with the presence of a favorable perfusion profile, the response to thrombolysis in terms of efficacy and safety in the sub-population of very old patients was not improved. A worse collateral supply in older compared to younger patients could partly explain this result. Indeed, the long term exposure to vascular risk factors could have reduced the permeability of collateral vessels [26].

Patients included in the present study had relatively high NIHSS at baseline, which, in combination with the old age could have increased the risk of poor prognosis. This result is in accordance with previous reports which suggested that a positive SPAN-100 (patient age + NIHSS at baseline ≥ 100) could predict no significant benefit of thrombolysis [27]. Unfortunately, the use of CTP parameters did not seem to modify this cut-off.

Swallowing disorders and delirium were significantly more frequent in the thrombolysed group. Swallowing disorders appeared to be related to stroke severity, and delirium to a side-effect of IV-tPA combined with the effect of age. Delirium could also have been favored by the cognitive frailty of these very elderly patients. In addition, the decubitus often imposed in the acute phase given the presence of a proximal intracranial occlusion probably increased these disorders. The trend to more frequent pulmonary infections in the thrombolysed group might have been the consequence of swallowing disorders in addition of agitation and altered awareness, with the risk of getting in a vicious circle. These factors might have contributed to impair the functional prognosis, increase mortality and interfere with the potential benefit of thrombolysis.

The high rate of hemorrhagic transformation observed in our population receiving IV-tPA (53 %) is in accordance with the rate predicted by the SPAN-100 [27]. Hemorrhagic transformation and symptomatic intracranial hemorrhage might also partly explain the absence of IV-tPA benefit in the present study as we showed that symptomatic intracranial hemorrhage tended to be associated with the mRS at three months and death in the first 7 days. However, in IST-3, symptomatic intracranial hemorrhage did not affect clearly the outcome at six months [23], and while thrombolysis was found to be associated with hemorrhagic transformation in bivariate analysis, it did not remain significant in multivariate analysis. Conversely, intracranial occlusion and stroke severity at 24 h were independent predictors of hemorrhagic transformation. The association between intracranial occlusion and hemorrhagic transformation has already been described [28] and might be related to the larger infarct size observed in this condition.

The results of this study should be interpreted cautiously due to several limitations. First, the retrospective design and the small sample size limits the power of statistical analysis. But the characteristics of our population are in line with previous studies and support the validity of our sample. Second, IV-tPA was administered until 4.5 h while some recommendations limit the use of this treatment to 3 h post-stroke onset in patients older than 80 y.o. [21]. However, treatment was initiated early in our sample (mean 168 ± SD 48 min) which might have limited the influence of the delay. Third, CTP imaging was not performed in non-thrombolysed patients, which did not allow direct comparisons between groups. Finally, the absence of evaluation of recanalization and/or reperfusion and final infarct size also limits the interpretation of results.

## Conclusion

IV-tPA administered early after IS onset in our sample of patients aged over 90 y.o. did not influence the functional prognosis whereas most of them had a favorable neuroimaging pattern. Moreover, hemorrhagic transformation and post-stroke complications were more frequent in thrombolysed patients, and symptomatic intracranial hemorrhage tended to worsen the functional outcome at three months and increase the rate of death in the first 7 days.

While the identification of additional predictors could be useful to improve the selection of patients suitable for IV-tPA, other revascularization techniques such as mechanical thrombectomy might be considered in this frail population in order to reduce hemorrhagic transformation, early clinical worsening, the vicious circle of post-stroke complications and extended duration of hospitalization. Further studies are needed to confirm those results.

## Additional file

Additional file 1: Table S1. Multivariate analysis of associations between pre-stroke and stroke characteristics, mRS at three months and death in the first 7 days (multiple linear regressions). (DOCX 22 kb)

## Abbreviations

ACA: Anterior cerebral artery
AF: Atrial fibrillation
ASPECTS: Alberta Stroke Program Early CT score
CBF: Cerebral blood flow
CBV: Cerebral blood volume
CT: Computed tomography
CTP: CT-perfusion
HI: Hemorrhagic infarction
IQCODE: Informant Questionnaire on Cognitive Decline in the Elderly
IS: Ischemic stroke
IV-tPA: Intravenous thrombolysis
M1: M2, M3, Segments of middle cerebral artery
mRS: Modified Rankin scale
MRI: Magnetic resonance imaging
MTT: Mean transit time
NIHSS: National Institute of Health Stroke Score
PCA: Posterior cerebral artery
PH: Parenchymal hemorrhage
SD: Standard deviation
TOAST: Trial of Org 10172 in Acute Stroke Treatment
VB: Vertebral-basilar arteries
