# Supplementary material for: The impact of intravenous thrombolysis on outcome of patients with acute ischemic stroke after 90 years old
Source: BMC Geriatr. 2016 Aug 25;16(1):156. doi: 10.1186/s12877-016-0331-1 (PMC5000473; doi:10.1186/s12877-016-0331-1)
Supplement: Additional file 1: Table S1. — Multivariate analysis of associations between pre-stroke and stroke characteristics, mRS at three months and death in the first 7 days (multiple linear regressions). (DOCX 22 kb) [file 12877_2016_331_MOESM1_ESM.docx]

**Additional file**

**Additional file 1**

**File format: .pdf**

**Title: Multivariate analysis of associations between pre-stroke and stroke characteristics, mRS at three months and death in the first 7 days (multiple linear regressions)**

|  | **mRS at three months** | | **Death ≤ 7 days** | |
| --- | --- | --- | --- | --- |
|  | **Estimate β (SE)** | **p** | **Estimate β (SE)** | **p** |
| **Hypercholesterolemia** | -0.6 (0.7) | 0.4 | -0.3 (0.2) | 0.1 |
| **Statins** | -0.4 (1.2) | 0.7 | 0.3 (0.3) | 0.3 |
| **Systolic blood pressure at baseline** | 0.004 (0.008) | 0.7 | -0.004 (0.003) | 0.2 |
| **NIHSS at baseline** | 0.03 (0.09) | 0.7 | -0.02 (0.02) | 0.4 |
| **NIHSS at 24 hours** | -0.008 (0.08) | 0.9 | 0.03 (0.02) | 0.1 |
| **Intracranial occlusion** | -0.08 (0.6) | 0.9 | -0.03 (0.2) | 0.9 |
| **Symptomatic intracranial hemorrhage** | 0.9 (1.1) | 0.4 | 0.3 (0.2) | 0.3 |
| **Cardioembolic stroke subtype (AF)** | 0.2 (0.9) | 0.8 | -0.05 (0.2) | 0.8 |

mRS: modified Rankin Scale, SE: standard error, NIHSS: National Institute of Health Stroke Scale, AF: atrial fibrillation
